# Classification of Known and Unknown Study Items in a Memory Task Using Single-Trial Event-Related Potentials and Convolutional Neural Networks

**DOI:** 10.3390/brainsci14090860

**Published:** 2024-08-26

**Authors:** Jorge Delgado-Munoz, Reiko Matsunaka, Kazuo Hiraki

**Affiliations:** Graduate School of Arts and Sciences, The University of Tokyo, Meguro-Ku, Tokyo 153-8902, Japan; matsunaka@ardbeg.c.u-tokyo.ac.jp (R.M.); khiraki@idea.c.u-tokyo.ac.jp (K.H.)

**Keywords:** long term memory, familiarity, electroencephalography, event-related potentials, convolutional neural networks

## Abstract

This study examines the feasibility of using event-related potentials (ERPs) obtained from electroencephalographic (EEG) recordings as biomarkers for long-term memory item classification. Previous studies have identified old/new effects in memory paradigms associated with explicit long-term memory and familiarity. Recent advancements in convolutional neural networks (CNNs) have enabled the classification of ERP trials under different conditions and the identification of features related to neural processes at the single-trial level. We employed this approach to compare three CNN models with distinct architectures using experimental data. Participants (*N* = 25) performed an association memory task while recording ERPs that were used for training and validation of the CNN models. The EEGNET-based model achieved the most reliable performance in terms of precision, recall, and specificity compared with the shallow and deep convolutional approaches. The classification accuracy of this model reached 62% for known items and 66% for unknown items. Good overall accuracy requires a trade-off between recall and specificity and depends on the architecture of the model and the dataset size. These results suggest the possibility of integrating ERP and CNN into online learning tools and identifying the underlying processes related to long-term memorization.

## 1. Introduction

Long-term memory can be classified into two types based on the type of information that must be retrieved: implicit and explicit. Implicit memory is related to procedures and the execution of specific tasks; it is not retrieved consciously and is mostly associated with skills or daily tasks that do not require relearning them to be performed. Explicit memory, in contrast, refers to the storage of factual and objective information through textbook learning or experiential memories, which are commonly acquired through rehearsal and must be retrieved consciously according to when such information is needed [1,2]. Explicit memory can be classified into episodic memory, which is related to specific events, and semantic memory, which is related to facts, concepts, and general knowledge.

Yonelinas [3] distinguished between two fundamental processes of long-term memory retrieval: recollection and familiarity. Recollection refers to the retrieval of details and contextual information regarding past events that surround the acquisition of new knowledge and memories. Alternatively, familiarity is based on the qualitative signal of an item and commonly refers to the capacity to identify an item by its name without any background or contextual information [4]. Distinguishing familiarity and recollection-based recognition remains a complex endeavor because these processes are associated with distinct brain regions and cognitive mechanisms [4].

Since the late 1990s, event-related neuroimaging has been employed to investigate the impact of different encoding strategies on subsequent memory retrieval, establishing an early foundation for the connection between familiarity and neural processes [5]. Several studies conducted since the beginning of the new millennium have used the event-related potential (ERP) technique as a tool for elucidating memory encoding and retrieval processes, emphasizing its high temporal resolution for studying the timing of the cognitive processes involved in memory [6]. Around the mid-2000s, advances in research yielded significant insights into the electrophysiological aspects of familiarity, with extensive studies exploring the FN400 component, an ERP response consistently linked to familiarity, and the old/new effect [7,8,9]. Recent evidence suggests that FN400 is sensitive to changes in contextual familiarity, indicating its role in the comparative evaluation of familiarity within a specific context [10]. The N400 component, typically associated with semantic processing, has also been implicated in familiarity assessments [9,10]. N400 appears to reflect an evaluation of absolute familiarity, independent of contextual information, introducing an additional layer of complexity to our understanding of the electrophysiological responses associated with familiarity. The last decade has witnessed a surge in the volume of studies dedicated to understanding familiarity and its neural substrates. This growing body of literature provides profound insights into the neural mechanisms underlying recognition memory, particularly focusing on the distinction between familiarity-based recognition and recollection-based source memory retrieval [11]. However, individual variations in reliance on familiarity versus recollection processes during recognition memory tasks have been highlighted, underscoring the non-uniformity of these processes across individuals [12].

One typical situation in which explicit semantic memory is applied is the memorization of a set of items related to a specific subject as part of an evaluation and assessment in different learning and education contexts. Such a set might contain a few items already known by the student that do not require restudy and others that the student may encounter for the first time or might not be recalled with sufficient confidence. This opens up the possibility of using the electrophysiological response as a biomarker to predict whether an item has been previously encountered or learned. Studies on the use of electrophysical recordings to quantitatively predict the degree of familiarity with a determined item during memorization tasks have been conducted. Fukuda and Woodman [13] conducted a study that specifically focused on predicting the recognition memory of an individual using electroencephalographic (EEG) signals. They collected EEG signals from participants engaged in recognition memory tasks. Their study focused on two key neural signals: the P3 component of the ERP and alpha power modulation. The latter, which refers to changes in the power of neural oscillations in the alpha frequency band (8–12 Hz), is assumed to be indicative of the inhibitory processes involved in gating sensory information and has been associated with memory performance. Furthermore, their study explored potential real-time interventions that could enhance memory performance based on predictions from EEG signals. Similarly, Khurana et al. [14] conducted a study aimed at investigating the combination of different EEG features and frequency bands to accurately predict word familiarity. The study involved recording EEG signals from participants as they were presented with words of varying familiarity levels. Both time- and frequency-domain features were extracted and analyzed. This study determined that a combination of specific EEG features and frequency bands resulted in an accurate prediction of word familiarity. This suggests a strong correlation between these EEG features and frequency bands and the familiarity ratings of words, further emphasizing the importance and potential of EEG features in predicting cognitive states.

Typically, ERP analysis is performed by epoching time-locked segments of the recorded EEG signal around the stimulus onset, referred to as trials, grouped into different categories depending on the condition of the task or the behavioral response of the participant, and thereafter averaging the signals from each group such that the signal-to-noise ratio of the recording is increased, and the components related to the neural process associated with the task are highlighted while canceling the irrelevant ones during the averaging operation [15,16,17]. Although this method usually achieves satisfactory results, the use of ERP in memory tasks has certain peculiarities that make it inadequate [18]. Memory paradigms have an implicit imbalance in the number of items that will be remembered or recognized, making the class with fewer trials not contain the necessary number of items required to identify components associated with the process. Moreover, the ability to encode new information is not perfect and varies from one individual to another [17]. This issue requires the analysis of ERP signals at the single-trial level, allowing the elucidation of existing individual variability [18].

To address these limitations, researchers have proposed various more sophisticated methods for single-trial analysis of EEG and ERP for classification at the single-trial level. Deep learning (DL) methodologies, including convolutional neural networks (CNNs) and recurrent neural networks, are of interest. In particular, CNNs, which have proven to be effective in analyzing image data, have achieved promising results in processing physiological signal datasets, such as epoched EEG recordings. Researchers have adapted these network architectures to process EEG data that involve time-series information representing neural activity over time [19,20,21,22]. CNNs have reportedly achieved a high classification accuracy in distinguishing between different types of stimuli and diagnoses. CNNs offer promising solutions for automated feature extraction and direct classification using raw EEG data [23]. This automation can overcome the challenges associated with feature engineering and selection that are inherent in traditional methods. Finally, the flexibility of neural networks in modeling nonlinear relationships and complex interactions within ERP data enables them to capture intricate patterns and dependencies. Their generalization capability enhances the applicability and reliability of single-trial analysis in diverse research settings, including rapid and on-the-fly analysis in experimental paradigms, such as brain–computer interfaces (BCIs) and neurofeedback applications [24]. DL methods have been successfully applied to classification problems involving time-locked stimuli in EEG recordings and ERP, such as the recognition of rhythm pattern perception [19], seizure detection [23], diagnosis of schizophrenia [20], neuromarketing [25], and attention levels during driving [26]. Using the proposed approach, we investigate the feasibility of integrating devices capable of recording and analyzing electrophysiological responses into digital and online learning tools to adjust the volume of learning items. The ERP response elicited by the presentation of each study item is used as the input of a CNN model to predict whether such an item has already been learned by the student and excluded from the learning list, or whether it is a newly encountered item that must be learned through repetition.

## 2. Materials and Methods

### 2.1. Participants

A group of 25 participants (*N* = 25) was recruited for the experiment. All participants were students at the University of Tokyo with an age range of 18–30 years (mean age = 20.96, SD = 3.06); 68% were male and 32% were female. Prior to the task, all participants were asked whether they were interested in geography and to decide whether to perform the task in Japanese or English; none of the participants reported any history of psychiatric disease. Data from four participants were excluded from the analysis owing to one of them reporting color blindness after the execution of the task, two of them owing to excessive signal artifacts, and one of them owing to corrupted behavioral data. Moreover, the data from three of the aforementioned participants correspond to individuals who did not perform the task in their native languages. The final analysis was performed on data from the remaining 21 participants (N = 21) who completed the task in Japanese (mean age = 20.04, SD = 2.13; 71.43% male, 28.57% female). The experiment was conducted in accordance with the Declaration of Helsinki and the ethics regulations of The University of Tokyo. Informed consent was obtained from all participants involved in the study, and they received monetary compensation for their cooperation.

### 2.2. Experimental Design

The experiment was designed using JSPsych, a JavaScript library specially designed to conduct psychology experiments capable of running on a web browser [27,28]. The experiment was conducted by presenting the task on a color 23-inch LCD computer screen in a soundproof room with a controlled temperature.

**Figure 1:**
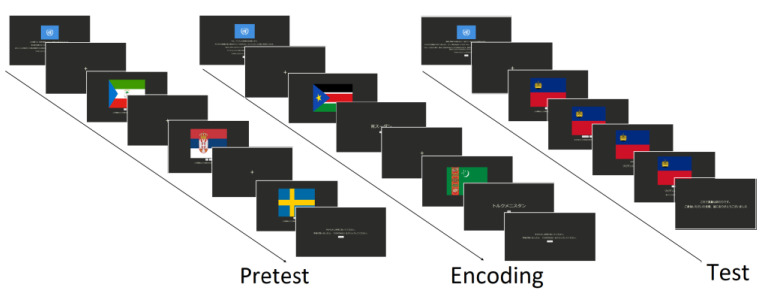
Structure of the experimental task. Participants were instructed to assess their previous knowledge during the pretest section, study the items during the encoding section, and evaluate the number of items they could remember in the test section. Only the pretest section is relevant for this article.

The experimental task comprised a full study session divided into three sections: pretest, encoding, and test, similar to the studies conducted by Fukuda and Woodman [13], Massol et al. [29], and Guo et al. [30]. The comprehensive structure of the task designed in this study is shown in Figure 1. The experiment began by providing a practice section to familiarize participants with the elements on the screen. Prior to the start of each section, a set of instructions was displayed on the screen and the participants were required to click on the START button to initiate the experiment. Each item presented on the screen comprised a display of a fixation cross for a variable time (800, 900, 1000, 1200, or 1300 ms) followed by the presentation of the visual stimuli with a prompt. The item set comprised the flags of United Nations member countries. For each participant, a subset of 60 items was randomly selected from the entire set of 193. During the pretest section, the prompt asked the participants whether they knew the name of the country to which the displayed flag belongs and required them to click the button corresponding to their desired answer (YES or NO). After the presentation of all the items, the screen displayed a message inviting the participants to take a short break before continuing with the next section. The encoding section involved practicing the entire list in a spaced repetitive manner. The set was divided into four subsets of 15 elements each, showing a flag for 1250 ms, followed by the corresponding country name. When the name of the country was displayed, the participant was invited to click the NEXT button to proceed to the next item. Once all 60 items had been studied, the participants were required to take a short break before studying the items three more times. Each time, the order of the item presentation was randomly changed. Finally, a test was performed to evaluate the number of items remembered after the encoding section. In this section, the flag of the country was displayed first, followed by a series of prompts on which the participants were asked to click a button whether they could remember the number of the country (NO or YES) and whether they felt confident about their answer (CONFIDENT, JUST GUESSING). Subsequently, the correct name of the country was displayed, and the final prompt asked the participants whether it was the name of the country they remembered. This sequence of prompts is conditioned by the first response. The participants were instructed to carefully read all the prompts and indications displayed on the screen during the task and blink only when a fixation cross appears on the screen.

### 2.3. EEG Signal Recording

EEG signals were collected using a Geodesic EEG system (Magstim EGI Inc., Eugene, OR, USA) comprising a Geodesic Sensor Net with 128 Ag/AgCl wet electrodes, a Net Amps 400 medical grade biosignal amplifier, and the Net Station software suite. The signals were recorded at a sampling rate of 250 Hz and referenced online to Cz. Before starting the signal recording, the impedance of each electrode was maintained under 10 KΩ.

**Figure 2:**
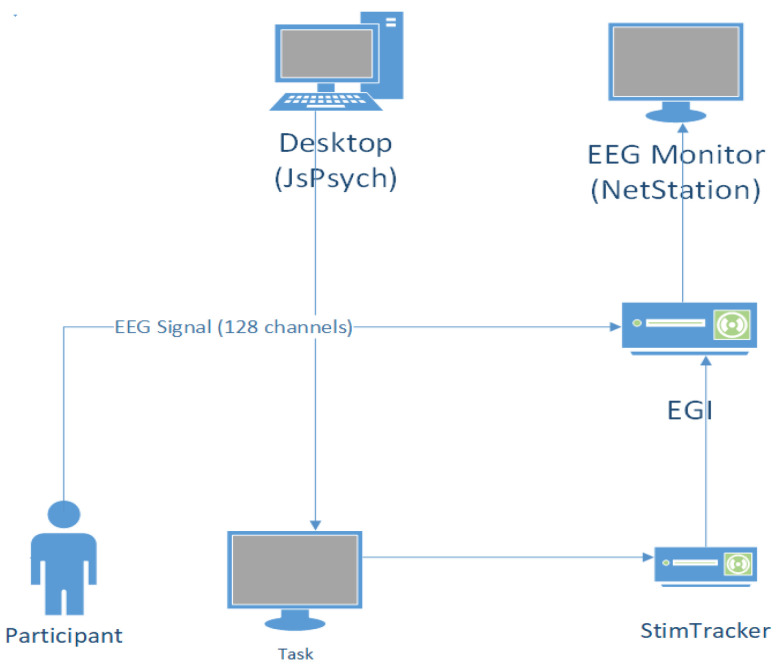
Experimental setup schematic. This diagram describes the apparatus and its use during the experimental task.

Events corresponding to item presentation on the screen were captured using a Cedrus StimTracker system (Cedrus Corporation, San Pedro, CA, USA). The stimuli were captured using StimTracker’s optical sensor attached to the LCD screen and synchronized with the EEG recording at the NET station via a parallel port connection between the StimTracker device and the amplifier. A schematic of the experimental setup is shown in Figure 2.

### 2.4. EEG Signal Preprocessing

EEG signal preprocessing was performed using MATLAB R2023a (Mathworks Inc., Natick, MA, USA). The recording archives were first imported using EEGLAB [31], an open-source toolbox for MATLAB used for the processing and analysis of neural data. Following the indications of Calbi et al. [32], the channels corresponding to the outer belt were removed prior to preprocessing to avoid the presence of muscle and movement artifacts, thereby reducing the initial number of electrodes from 128 to 110. Subsequently, the entire EEG recording archives for each participant were segmented into three parts corresponding to different sections of the task.

**Figure 3:**
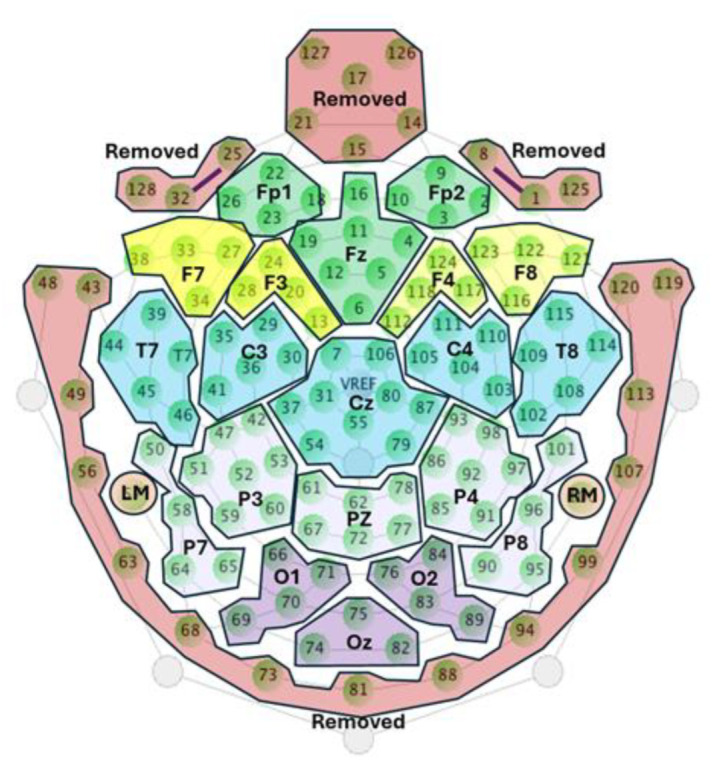
Clustering of electrodes for channel number reduction. Channels marked in red were removed prior to signal preprocessing.

Each recording segment was processed as follows: Signals were filtered using a notch filter at 50 Hz to remove power line noise, and a bandpass filter between 0.1 and 30 Hz to eliminate DC offset and EMG artifacts. The EEG channels were re-referenced offline to the average of the left and right mastoids, allowing reconstruction of the Cz channel. Subsequently, the dataset was downsampled to 128 Hz to comply with the input requirements for generating the CNN models. The remaining channels were clustered according to the 10–20 system to reduce the number of channels from 110 to 22 [32,33,34]. The criteria for performing this clustering were to select the channel labeled according to the 10–20 system as a reference and to average it with adjacent channels. Channels that show excessive artifacts were excluded from their corresponding clusters, and only the cleanest channels were used. Figure 3 shows the channels that were initially removed and the clustering of different electrode groups to reduce the number of channels.

### 2.5. ERP Processing

**Figure 4:**
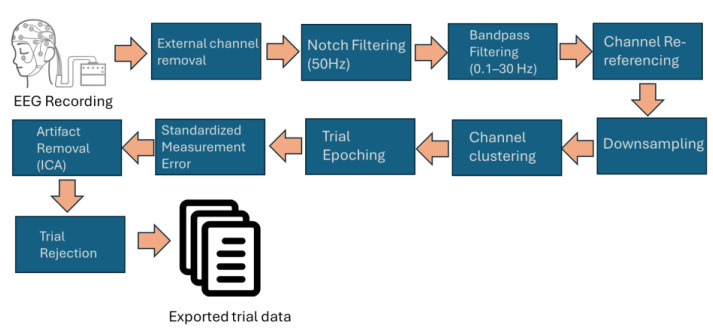
EEG processing pipeline block diagram.

The ERPLAB plugin [35] of EEGLAB was used to identify the ERP responses associated with each stimulus. Once the data were preprocessed, event information containing the behavioral responses of the participants and class information were imported into the datasets corresponding to each participant. The data were epoched selecting a time window between −200 and 1000 ms from the stimulus presentation and baseline corrected at 200 ms prior to the stimulus presentation. Using a standardized measurement error tool [36], channels Fp1 and Fp2 were rejected because of the presence of excessive noise in the majority of the participants’ data, reducing the number of channels from 22 to 20. Next, an artifact correction operation was performed. First, eye blink artifacts were removed using ICA and the IClabel tool from EEGLAB. Subsequently, ERP segments that contained peak-to-peak artifacts and step-like artifacts were rejected using the artifact removal tool from ERPLAB. A summarized diagram of the complete EEG signal processing pipeline utilized in this study is shown in Figure 4. All the trials were exported to each individual CSV file, starting from stimulus onset to the duration of the previously defined trial (1000 ms/128 data points). Each CSV file comprises a two-dimensional array, where the number of rows corresponds to that of EEG channels, and that of columns corresponds to that of samples in the trial [37].

### 2.6. CNN Architecture Description

#### 2.6.1. EEGNet

**Figure 5:**
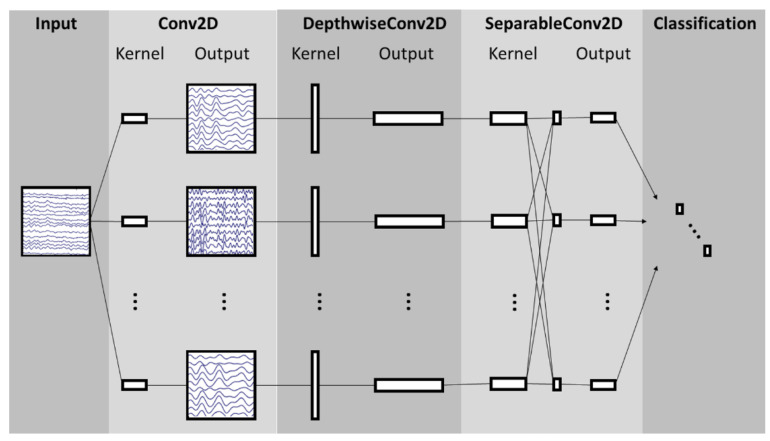
EEGNet architecture [38].

EEGNet is a CNN architecture specifically designed for EEG signal processing in BCIs [38]. This architecture comprises a temporal convolution layer, which learns frequency filters; a depthwise convolution layer, which learns specific spatial filters connected to each feature map individually; a separable convolution layer, which combines depthwise convolution and learns a temporal summary for each feature map individually; and a pointwise convolution layer, which learns to mix the feature maps together. Figure 5 shows the structure of the NN architecture. EEGNet-based models can be customized by modifying parameters C, T, F1, and F2, where C denotes the number of channels; T denotes the number of timepoints or samples in each trial; F1 denotes the number of temporal filters; D denotes the number of spatial filters; F2 denotes the number of pointwise filters, defined as F1 × D; and N denotes the number of classes. In addition, the filter size for the first layer is defined as half the number of samples. The notation EEGNET-8,2 refers to the default parameters of the architecture, with F1 = 8 and D = 2.

#### 2.6.2. Deep Convolutional Neural Network (DeepConvNet)

**Figure 6:**
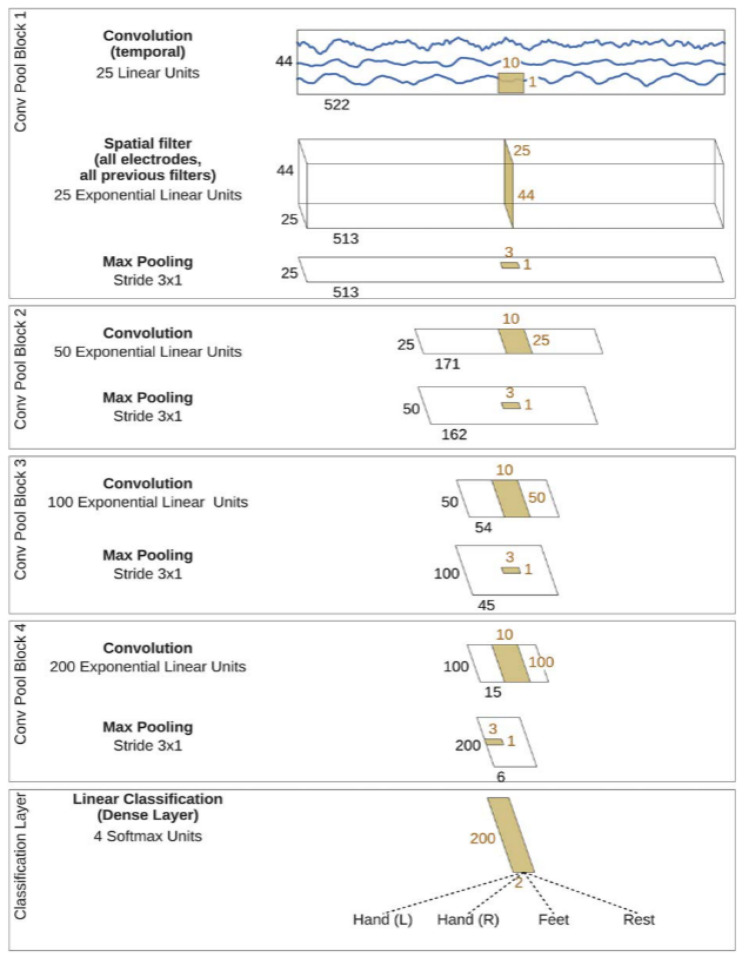
DeepConvNet architecture for multiclassification problem [37].

The deep convolutional neural network (DeepConvNet) used in this study was conceived as a model capable of extracting a wide range of features without being limited to specific types [37]. This generic architecture aims to achieve competitive accuracies with minimal expert knowledge and demonstrates the potential of standard CNNs for brain-signal decoding tasks. The architecture comprised four convolution-max-pooling blocks, with the first block specially designed to handle the EEG input. It used two layers in the first convolutional block to handle several input channels, and the second layer performed spatial filtering across electrode pairs. Exponential linear units were used as activation functions. Design choices were evaluated against alternative options, such as rectified linear units. The basic architecture of the multiclass classification problem is illustrated in Figure 6.

#### 2.6.3. Shallow Convolutional Neural Network (ShallowConvNet)

**Figure 7:**
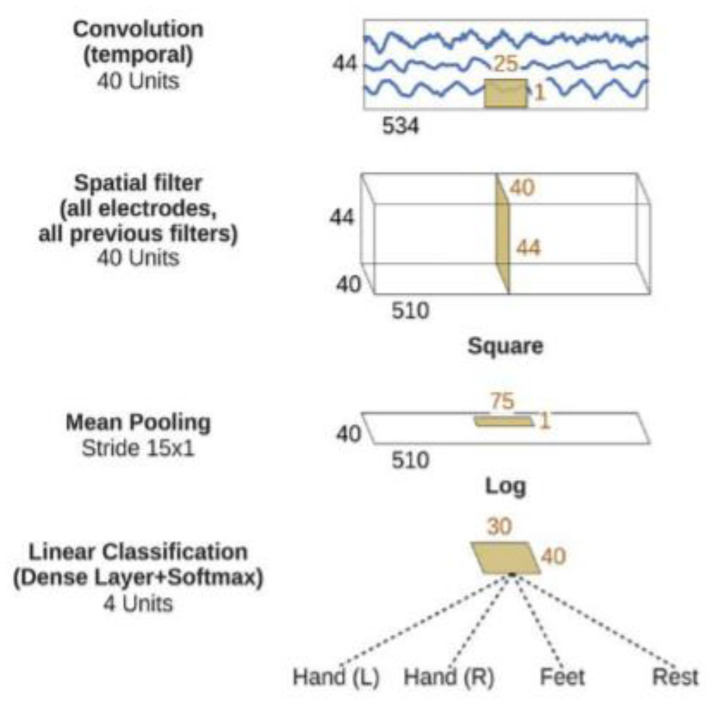
ShallowConvNet architecture for multi-classification problems [37].

The (ShallowConvNet) architecture was inspired by the FBCSP pipeline and optimized to decode band power features [37]. This architecture comprises temporal convolution and spatial filtering akin to FBCSP, with larger kernel sizes to allow for a broader range of transformations. The following steps are squaring nonlinearity, mean pooling, and logarithmic activation functions, mirroring the trial log-variance computation in FBCSP. Unlike FBCSP, ShallowConvNet integrates all the computational steps into a single network, enabling joint optimization. In addition, it incorporates multiple pooling regions within one trial, facilitating the learning of the temporal structures of band power changes, which have been shown to improve classification accuracy. The ShallowConvNet architecture is illustrated in Figure 7.

### 2.7. Pretraining of the Models

The data used to train the CNN models corresponded to the trials performed in the pretest section, for a total of 1260 trials. The artifact rejection operation resulted in 1153 usable and 107 rejected trials. The operating data were separated into 80% of the trials for use as training data, and the remaining 20% were used as testing data. The architectures for the models were implemented using an online library written in Python using the TensorFlow and Keras frameworks [37,39]. The library was slightly modified to use a sigmoid activation function, which always returns a value between 0 and 1 [40] and is more suited for binary classification, instead of softmax in the last layer, and imported into the Google Colaboratory (Colab) environment for training and validation. This environment runs on an Intel^®^ Xeon^®^ CPU at 2.30 GHz, an NVIDIA Tesla T4 GPU accelerator, and 12.72 GB of RAM memory. 

After the data were imported into the development environment, each channel was normalized such that the signal amplitude ranged from 0 to 1. Because the input data proportion of trials labeled as “Unknown” with respect to those labeled as “Known” is approximately 2.7:1, the training set was augmented using the RandomOverSampler function from the imblearn library, using maximization of the minoritarian class in the set as the sampling strategy. This resulted in 1342 trials for the training set. Additionally, the same number of trials as in the testing set were randomly extracted from the training set as validation data, leaving 20% of the original data for validation, and 20% for testing. The models were trained using an ADAM optimizer with a learning rate of 0.0001 and a binary cross-entropy loss function. Finally, a checkpoint callback was implemented to store the weights that provided the lowest result for the loss function at the end of the training. Model pretraining was performed using 500 epochs, a batch size of 64 for EEGNET, a batch size of 16 for DeepConvNet, and a batch size of 32 for ShallowConvNet. A summary of the hyperparameters used for training the models is presented in Table 1.

Table 2 shows the summary of the model architectures generated during this step. Finally, the models with the best overall performance based on the overall accuracy and loss function across the training and true positive rate for each class assessed by their respective confusion matrices were selected, and their respective weights were stored externally for further cross-validation.

### 2.8. Model Assessment

Each architecture was validated using stratified 10-fold cross-validation. For EEGNET and DeepConvNet, folds were generated using the weights obtained in the pretraining step, generating one model per fold for each architecture. The test data were used in each model to generate metrics per fold.

For the ShallowConvNet architecture, cross-validation was performed by loading the weights generated in the pretraining step and performing training and testing per fold in a single step without exporting the generated models in each fold.

The data used for the assessment were the augmented training set and the testing set from the previous steps. The models generated per fold were used to obtain the overall accuracy of the model, the receiver operating characteristic (ROC) curve, and metrics derived from their corresponding confusion matrices (sensitivity and specificity).

## 3. Results

### 3.1. Pretraining Results

**Figure 8:**
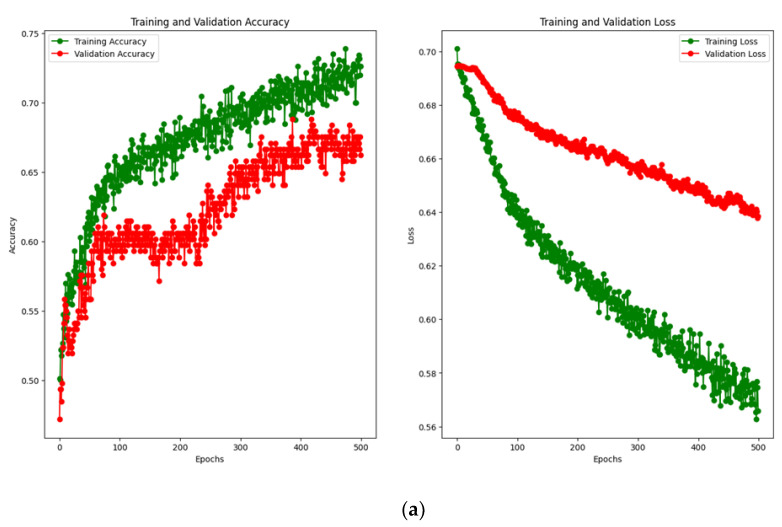

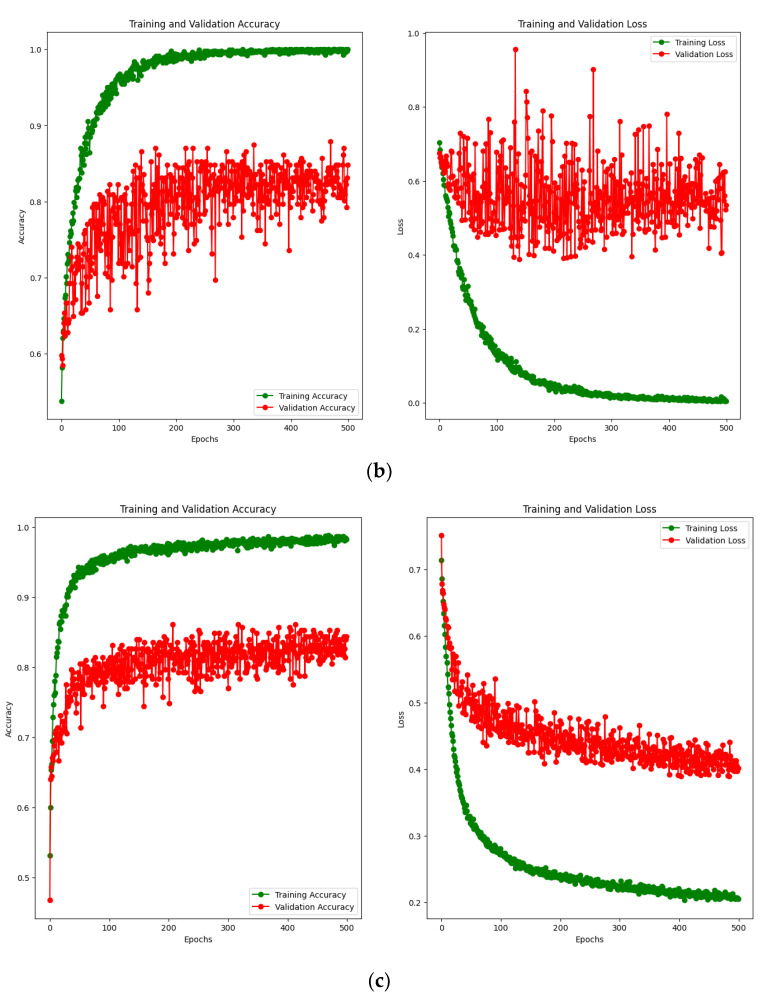
Accuracy and loss function plots for pre-trained models: (**a**) EEGNet; (**b**) DeepConvNet; and (**c**) ShallowConvNet. The early stop checkpoint allowed storage of the weights that provided the minimum loss function result and maintained them at the end of the training to avoid overfitting.

**Figure 9:**
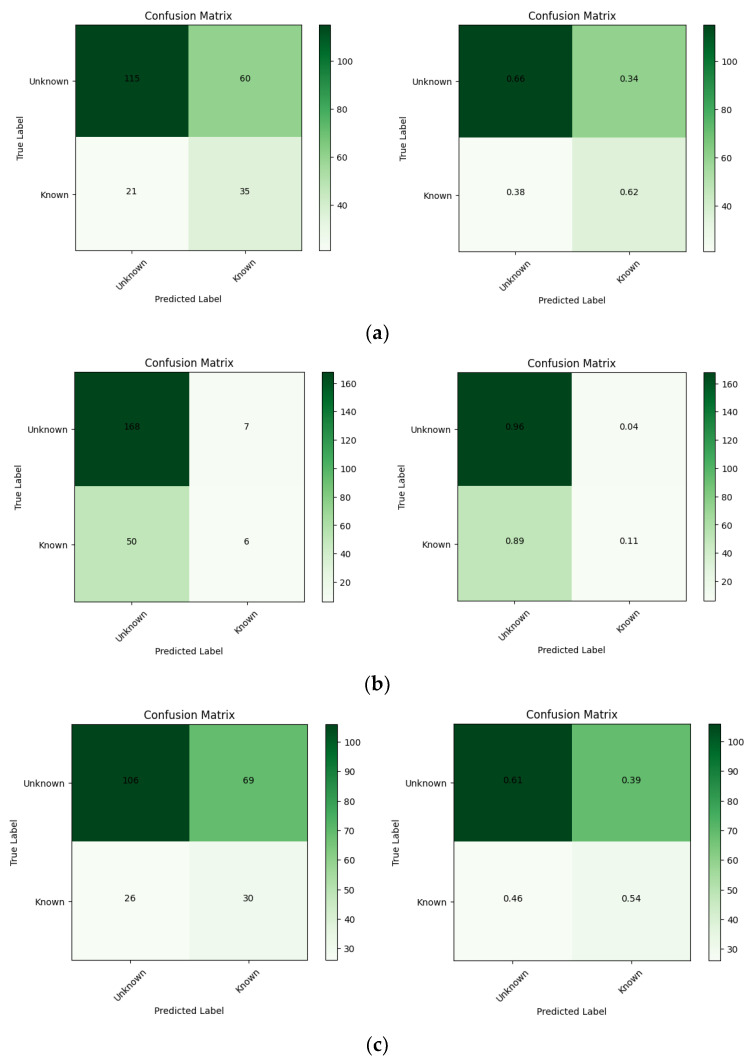
Confusion matrices for pre-trained models on test data: (**a**) EEGNet; (**b**) DeepConvNet; and (**c**) ShallowConvNet. The false positive rate for DeepConvNet and ShallowConvNet is significantly higher than that for EEGNet. The left panel shows the non-normalized matrix and the right one shows the normalized matrix.

**Figure 10:**
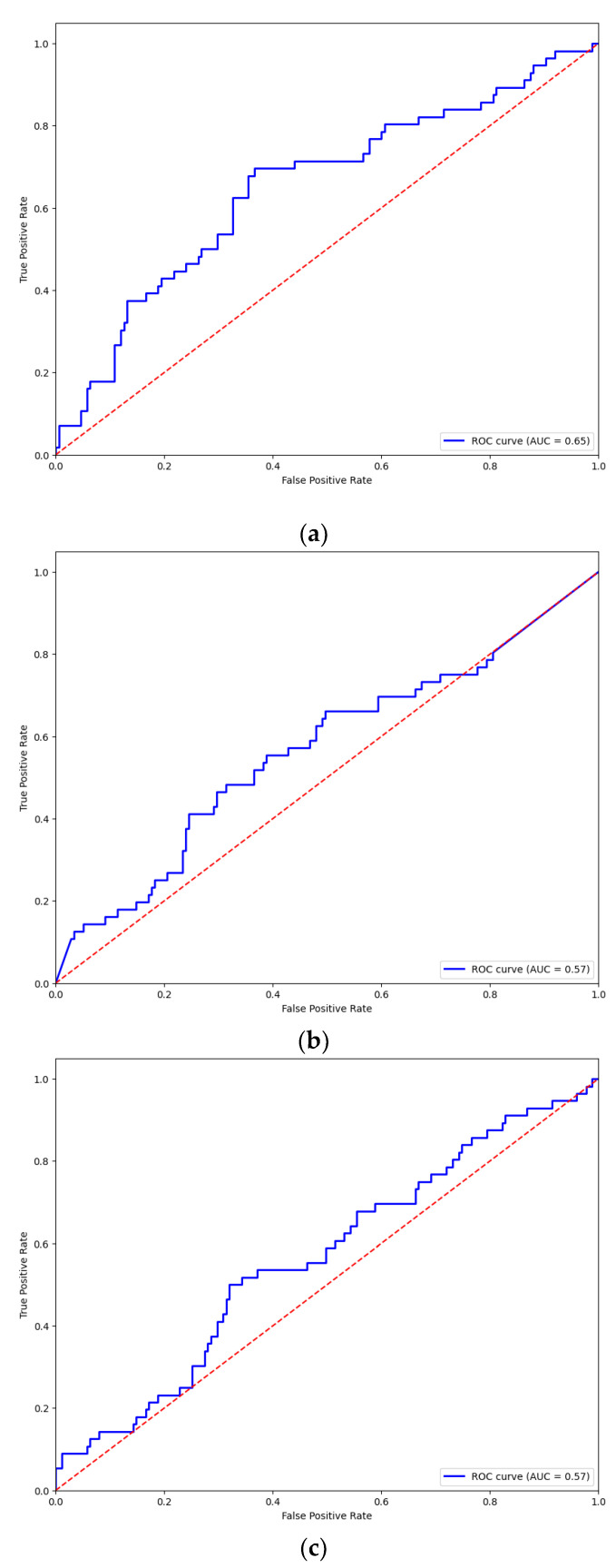
ROC curves for pre-trained models: (**a**) EEGNet; (**b**) DeepConvNet; and (**c**) ShallowConvNet. All three architectures show predictions above chance.

The performance of the pre-trained model for each architecture was assessed in terms of the overall accuracy and loss function during training, and the resulting plots are displayed in Figure 8. Table 3 summarizes the results of the pre-training including the metrics from the confusion matrix and ROC curve for each architecture. Figure 9 shows the confusion matrix for the prediction of each model based on the test data. The ROC curves for the pre-trained models are shown in Figure 10.

The EEGNet model provides the highest overall accuracy among the three architectures; however, the DeepConvNet and ShallowConvNet models outperformed EEGNet in terms of specificity or accurate prediction of trials belonging to the unknown class. From the confusion matrix, a higher specificity index in the unknown class is achieved in these models to the detriment of sensitivity, making them less reliable than EEGNet for predicting trials to belong to the known class. In addition, despite not having significantly higher ratings, EEGNet provided less susceptibility to overfitting and more consistent results for both classes. In addition, the areas under the curves (AUCs) suggest that the three models provide similar degrees of separation, which are slightly higher than random guessing, EEGNet being the architecture with the greatest AUC.

**Table 3:** Summary of metrics for pre-trained models.

Model	Overall Accuracy	ROC AUC	Sensitivity	Specificity
EEGNET	0.650	0.650	0.625	0.657
DeepConvNet	0.550	0.57	0.107	0.943
ShallowConvNet	0.610	0.57	0.530	0.606

### 3.2. Cross-Validation Results

**Figure 11:**
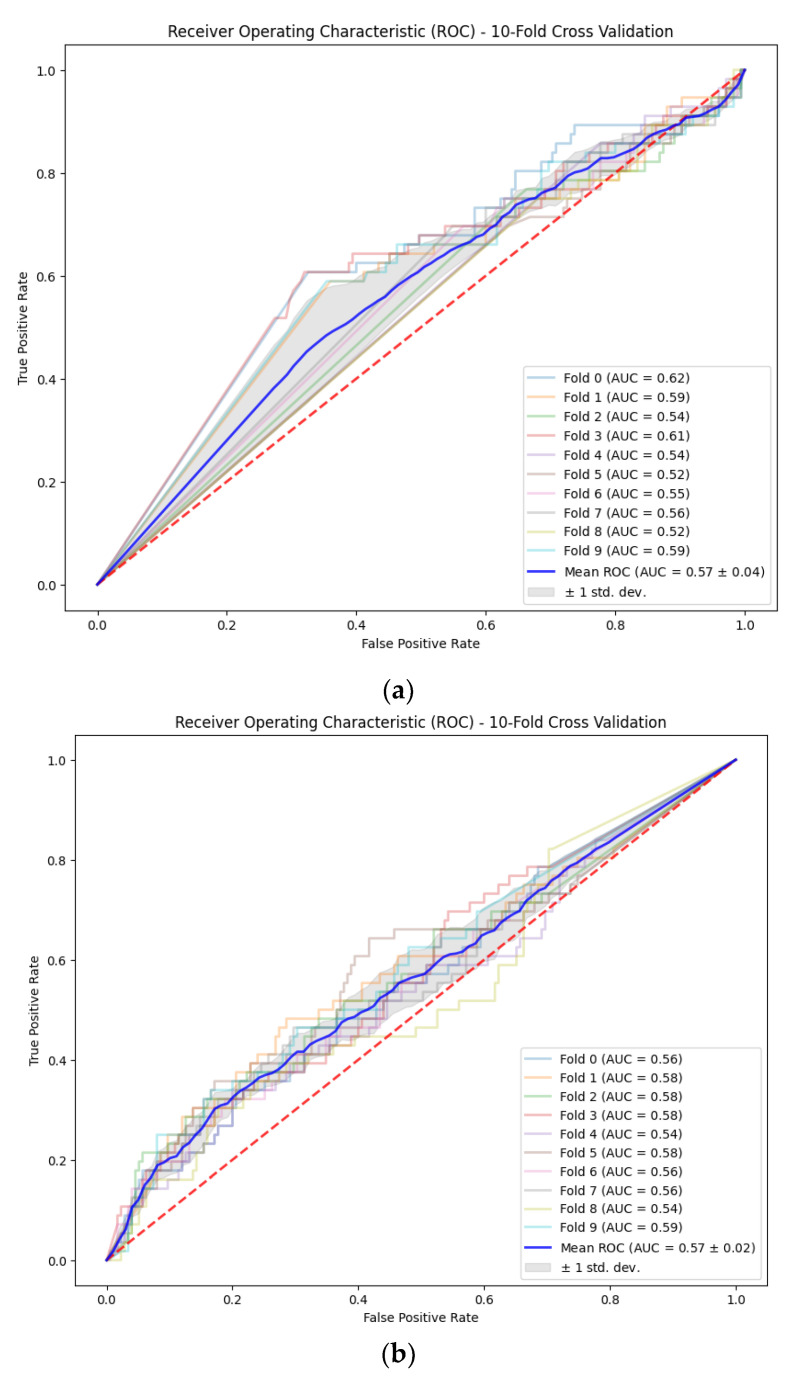

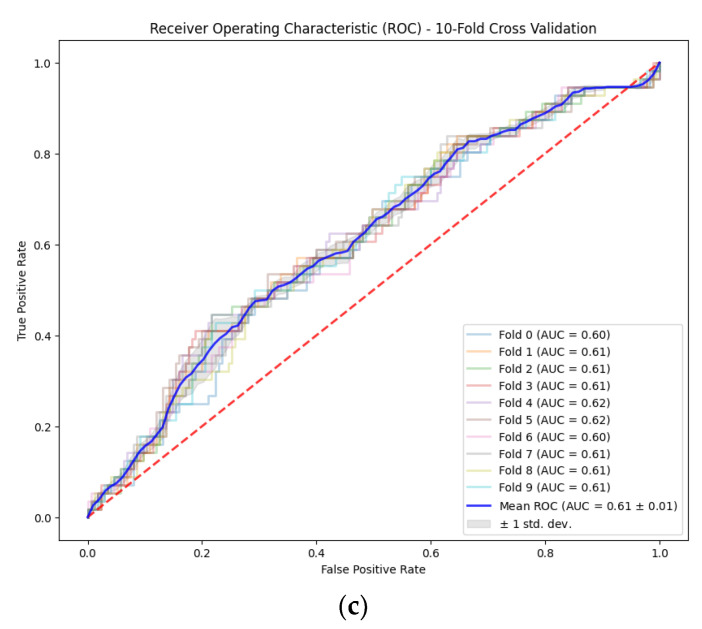
ROC curves for each model during cross-validation: (**a**) EEGNet; (**b**) DeepConvNet; and (**c**) ShallowConvNet. Results are consistent with those obtained from the pre-trained models.

To assess the general performance of each CNN architecture, the metrics derived from the ROC curves and the confusion matrix metrics calculated for each fold are summarized in Table 4. Figure 11 shows the corresponding ROC curves for each model architecture in each of the folds.

The metrics obtained from the cross-validation provide results that are partially consistent with those obtained from the pre-trained models since EEGNet and ShallowConvNet show smaller specificity indexes in cross-validation, but the indexes for EEGNet are close to the ones from ShallowConvNet. ShallowConvNet shows a greater sensitivity index than EEGNet and DeepConvNet. The AUC calculated after the cross-validation is very similar among all architectures, but is slightly greater for ShallowConvNet.

## 4. Discussion

In this study, we carried out an experiment aimed at using the stimuli-locked ERP recordings as biomarkers to predict if a study item, in this case, represented by a country flag, has been previously learned. This study aims to leverage the use of EEG and the previous knowledge on ERP and neural processes associated with the old/new effect, familiarity, and computational advantages provided by CNN [41].

Despite the proven efficacy of classical machine learning algorithms for EEG recording classification, CNN has gained popularity in diagnostic contexts owing to its capability to analyze complex nonlinear relationships between EEG inputs and output classification labels. Additionally, CNN is able to capture temporal data, autonomously extract features, handle large datasets efficiently, and adapt to diverse EEG applications [38]. However, the accuracy and robustness of CNN models mostly rely on the utilization of large training datasets to provide a proper generalization, which is often not available in the case of EEG signal recordings. This is added to the black-box nature of the model, which often provides an output without comprehending the exact underlying neural mechanisms. Despite the latter limitation, we believe that the use of CNN is still a promising choice given that classical paradigms used to elucidate neural processes associated with memory through ERP analysis are not aimed at understanding specific ERP components but use experimental manipulations instead to identify components of interest. This is stated under the belief that some processes will be engaged in some conditions but not in others [17].

### 4.1. Comparison with Previous Studies

Previous studies that have used the same CNN architectures applied in our study (EEGNet, DeepConvNet, and ShallowConvNet) and enhancements to them have proven the efficacy of these models as EEG signal classifiers. Regarding the EEGNet architecture, studies such as the one carried out by Zhao et al. [42] achieved up to 92.59% average accuracy for the diagnosis of Autism Spectrum Disorder using an enhanced version of this architecture. Zhang et al. [43] achieved up to a 75% recall rate in an RSVP study aimed at identifying the P300 component. A study by Xia et al. [44] achieved up to 90% overall accuracy in the classification of sleep. Fu et al. [45] reported up to 82% overall accuracy in the classification of Motor Imagery Tasks.

Regarding Deep and Shallow ConvNet architectures, studies such as the one carried out by Altan et al. [46] reported an overall accuracy of up to 90.3% for Motor imagery tasks. The study by Jo et al. [47] related to the prediction of visual memorability is of special interest to us because the procedure followed by the authors is quite similar to that proposed in this manuscript. They obtained 66.1% and 64% using ShallowConvNet and DeepConvNet, respectively, which are close to the results obtained in our study.

Based on the results obtained from the testing and validation of the different CNN models used in the current study, it can be observed that the output metrics from our study are low compared with the ones obtained by the same models in other domains, but not far from what was achieved by similar studies such as the ones mentioned in [43,47]. Additionally, the overall accuracy in DeepConvNet and ShallowConvNet can reach higher values than in EEGNet, as can be observed in Table 4. However, DeepConvNet fails to provide a reliable sensitivity rate; much lower levels of predicted items on the known class were also observed in the DeepConvNet and ShallowConvNet-based studies by Jo et al. [47]. On this foundation, we conclude that the EEGNet and ShallowConvNet architecture provide a better understanding of the underlying neural mechanisms associated with explicit memory and familiarity than DeepConvNet from an electrophysiological perspective. Since the problem we are addressing in this study assumes that both classes (known and unknown) are equally important, we consider that a predictive tool based on EEGNet would be more reliable in a real-life context, in which a digital tool is used to assist the study of a set of items. The reason why the DeepConvNet architecture have a low sensitivity rate compared to EEGNet and DeepConvNet despite being all CNN models, may lie in their design and architecture. As mentioned in the Materials and Methods section, the reliability of EEGNet might be due to the flexibility and adaptability provided by its parametrized structure, which is applicable to different datasets and accommodates a multiple number of channels, time samples, and spatial filters [44]. On the other hand, the architectures of DeepConvNet and ShallowConvNet do not rely much on parameters from input data and emphasize more the number of convolutional layers and the use of operations that are similar to filtering in FBSCSP [46]. However, it is important to notice that the architecture of ShallowConvNet is relatively simple and can outperform DeepConvNet despite its number of convolutional layers.

### 4.2. Limitations of the Study

One of the most important limitations at the time of carrying out the study lies in the nature of the tasks used to perform ERP analysis in memory. According to Wilding [17], the challenge in the design of a memory experiment consists of recruiting enough participants to have as much statistical significance as possible and collecting enough trials for each class of interest to guarantee such significance while making the participants able to complete the task and remain engaged in it. This limitation associated with classical ERP analysis also seems to have repercussions when using other methods, such as DL. Initially, the experiment was designed based on the number of participants and trials necessary to perform ERP analysis using the grand averaging method [15,16,17]. Even though the number of participants was reduced, the obtained results allowed us to infer that using a larger dataset and a more balanced number of trials corresponding to each category can sharply increase the accuracy and reliability of the models.

One of the most important challenges that must be overcome when implementing DL-based solutions is the amount of data available for training. Using EEG data to train models is limited by the reduced number of freely available datasets compared with other types of data used for training them, such as images, and the specificity of the collected data regarding the paradigm and purpose, owing to the different responses associated with multiple neural processes. Nevertheless, the models used in this study can still perform predictions with acceptable accuracy, which is consistent with the results obtained in other studies that use CNNs to perform classification based on EEG signals in different contexts and using a rather limited amount of training data [21,25,26,43,47]. This could be observed in the AUCs, which are similar in all three models and provide a prediction rate slightly above chance. The problem of the limited amount of training data was partially overcome by oversampling the existing data, which was intended mainly to increase the number of trials from the minority class to make the training dataset more balanced, hence improving the sensitivity and specificity indexes. However, we believe that the use of real human-balanced data will contribute to increasing the overall performance of the model and provide a more reliable prediction. It is important to mention that given the black-box nature of the convolutional neural network approach, the reduced dataset used for training, and the individual differences in brain anatomy and cognitive strategies across participants, the training data may contain noise and subsequently compromise the accuracy of the model.

## 5. Conclusions

In this study, we proposed a method to assess familiarity and new/old effects using a DL approach as a surrogate to the traditional grand average method used to identify such phenomena. To the best of our knowledge, this is one of the few attempts available to assess the performance of architectures tested in other paradigms that involve the use of EEG and ERP in a memory task to predict whether a study item has been previously learned. DL approaches are a promising solution for performing a single-trial analysis of ERP data using classification results and features learned through convolutional layers. The reduced amount of data available for training and the difficulty in obtaining balanced amounts of trials for different classes influence the performance of the models. Future studies on this topic will focus on improving the accuracy of the model by collecting greater amounts of training data, customizing and combining the architectures of different models, and performing transfer learning from previously trained models to obtain more accurate prediction outputs. We expect that the use of CNN models will allow the creation of online tools capable of recording physiological activity in real time and assessing the learning process based on the neural responses of students.

## Figures and Tables

**Table 1:** Hyperparameters are used to train the different models.

Hyperparameter	Values		
	EEGNet	DeepConvNet	ShallowConvNet
Kernel length	64	64	64
F1	8	N/A	N/A
D	2	N/A	N/A
F2	16	N/A	N/A
Optimizer	ADAM	ADAM	ADAM
Loss Function	Binary Crossentropy	Binary Crossentropy	Binary Crossentropy
Learning rate	1 × 10^−4^	1 × 10^−4^	1 × 10^−4^
Batch Size	64	16	32
Regularization of L1	1 × 10^−5^	N/A	1 × 10^−5^
Regularization of L2	1 × 10^−5^	N/A	1 × 10^−5^
Dropout Rate	0.2	0.2	0.2
Epochs	500	500	500

**Table 2:** Summary of the CNN architectures in the development environment: (a) EEGNet; (b) DeepConvNet; and (c) ShallowConvNet.

(a) EEGNet			(b) DeepConvNet			(c) ShallowConvNet		
Layer (Type)	Output Shape	Param #	Layer (Type)	Output Shape	Param #	Layer (Type)	Output Shape	Param #
input_3 (InputLayer)	[(None, 20, 128, 1)]	0	input_4 (InputLayer)	[(None, 20, 128, 1)]	0	input_1 (InputLayer)	[(None, 20, 128, 1)]	0
			conv2d_15 (Conv2D)	(None, 20, 124, 25)	150			
conv2d_2 (Conv2D)	(None, 20, 128, 8)	512	conv2d_16 (Conv2D)	(None, 1, 124, 25)	12,525			
			batch_normalization_12 (BatchNormalization)	(None, 1, 124, 25)	100	conv2d (Conv2D)	(None, 20, 116, 40)	560
batch_normalization_6 (BatchNormalization)	(None, 20, 128, 8)	32	activation_15 (Activation)	(None, 1, 124, 25)	0			
			max_pooling2d_12 (MaxPooling2D)	(None, 1, 62, 25)	0			
depthwise_conv2d_2 (DepthwiseConv2D)	(None, 1, 128, 16)	320	dropout_12 (Dropout)	(None, 1, 62, 25)	0	conv2d_1 (Conv2D)	(None, 1, 116, 40)	32,000
			conv2d_17 (Conv2D)	(None, 1, 58, 50)	6300			
batch_normalization_7 (BatchNormalization)	(None, 1, 128, 16)	64	batch_normalization_13 (BatchNormalization)	(None, 1, 58, 50)	200			
			activation_16 (Activation)	(None, 1, 58, 50)	0	batch_normalization (Batch Normalization)	(None, 1, 116, 40)	160
activation_4 (Activation)	(None, 1, 128, 16)	0	max_pooling2d_13 (MaxPooling2D)	(None, 1, 29, 50)	0			
			dropout_13 (Dropout)	(None, 1, 29, 50)	0			
average_pooling2d_4 (AveragePooling2D)	(None, 1, 32, 16)	0	conv2d_18 (Conv2D)	(None, 1, 25, 100)	25,100	activation (Activation)	(None, 1, 116, 40)	0
			batch_normalization_14 (BatchNormalization)	(None, 1, 25, 100)	400			
spatial_dropout2d_4 (SpatialDropout2D)	(None, 1, 32, 16)	0	activation_17 (Activation)	(None, 1, 25, 100)	0			
			max_pooling2d_14 (MaxPooling2D)	(None, 1, 12, 100)	0	average_pooling2d (AveragePooling2D)	(None, 1, 12, 40)	0
separable_conv2d_2 (SeparableConv2D)	(None, 1, 32, 16)	512	dropout_14 (Dropout)	(None, 1, 12, 100)	0			
			conv2d_19 (Conv2D)	(None, 1, 8, 200)	100,200			
batch_normalization_8 (BatchNormalization)	(None, 1, 32, 16)	64	batch_normalization_15 (BatchNormalization)	(None, 1, 8, 200)	800	activation_1 (Activation)	(None, 1, 12, 40)	0
activation_5 (Activation)	(None, 1, 32, 16)	0	activation_18 (Activation)	(None, 1, 8, 200)	0			
average_pooling2d_5 (AveragePooling2D)	(None, 1, 4, 16)	0	max_pooling2d_15 (MaxPooling2D)	(None, 1, 4, 200)	0			
spatial_dropout2d_5 (SpatialDropout2D)	(None, 1, 4, 16)	0	dropout_15 (Dropout)	(None, 1, 4, 200)	0	dropout (Dropout)	(None, 1, 12, 40)	0
flatten (Flatten)	(None, 64)	0	flatten_3 (Flatten)	(None, 800)	0	flatten (Flatten)	(None, 480)	0
dense (Dense)	(None, 2)	130	dense_3 (Dense)	(None, 2)	1602	dense (Dense)	(None, 2)	962
sigmoid (Activation)	(None, 2)	0	activation_19 (Activation)	(None, 2)	0	activation_2 (Activation)	(None, 2)	0
Total params: 1634 (6.38 KB)			Total params: 147,377 (575.69 KB)			Total params: 33,682 (131.57 KB)		
Trainable params: 1554 (6.07 KB)			Trainable params: 146,627 (572.76 KB)			Trainable params: 33,602 (131.26 KB)		
Non-trainable params: 80 (320.00 Byte)			Non-trainable params: 750 (2.93 KB)			Non-trainable params: 80 (320.00 Byte)		

**Table 4:** Metrics of ROC and confusion matrix per fold.

Model	Fold	Overall Accuracy	ROC-AUC	Sensitivity	Specificity
EEGNET	0	0.689	0.624	0.732	0.394
	1	0.674	0.589	0.750	0.309
	2	0.799	0.539	0.857	0.120
	3	0.709	0.615	0.696	0.451
	4	0.716	0.537	0.929	0.046
	5	0.739	0.522	0.857	0.126
	6	0.701	0.552	0.875	0.126
	7	0.649	0.562	0.875	0.189
	8	0.627	0.524	0.893	0.097
	9	0.701	0.594	0.750	0.343
	Mean	0.700	0.566	0.821	0.220
	SD	0.047	0.037	0.081	0.142
DeepConvNet	0	0.978	0.562	0.054	0.971
	1	0.993	0.583	0.286	0.869
	2	0.970	0.575	0.143	0.954
	3	0.985	0.577	0.107	0.966
	4	0.985	0.543	0.054	0.966
	5	0.993	0.583	0.143	0.937
	6	0.978	0.562	0.125	0.960
	7	0.985	0.559	0.250	0.869
	8	0.970	0.537	0.000	0.977
	9	0.978	0.586	0.018	0.971
	Mean	0.981	0.567	0.118	0.944
	SD	0.008	0.017	0.094	0.041
ShallowConvNet	0	0.815	0.598	0.732	0.429
	1	0.956	0.612	0.839	0.354
	2	0.978	0.612	0.839	0.320
	3	0.955	0.610	0.821	0.343
	4	0.963	0.615	0.839	0.320
	5	0.985	0.619	0.875	0.223
	6	0.993	0.602	0.804	0.314
	7	0.978	0.610	0.857	0.257
	8	0.993	0.606	0.875	0.251
	9	0.978	0.609	0.857	0.234
	Mean	0.815	0.598	0.732	0.429
	SD	0.956	0.612	0.839	0.354

## Data Availability

Data collected from the experiment are available upon request to the corresponding author. The code containing the experimental task can be accessed at https://github.com/joardemu85/JsPsych_Tasks/tree/main/04_Familiarity_Flags (accessed on 30 July 2024) The code used for analysis can be accessed at https://github.com/joardemu85/AMT_Code (accessed on 30 July 2024).
